# An Online Mindfulness-Based Cognitive Behavioral Therapy Intervention for Youth Diagnosed With Major Depressive Disorders: Protocol for a Randomized Controlled Trial

**DOI:** 10.2196/11591

**Published:** 2019-07-29

**Authors:** Paul Ritvo, Zafiris J Daskalakis, George Tomlinson, Arun Ravindran, Renee Linklater, Megan Kirk Chang, Yuliya Knyahnytska, Jonathan Lee, Nazanin Alavi, Shari Bai, Lillian Harber, Tania Jain, Joel Katz

**Affiliations:** 1 School of Kinesiology and Health Science York University Toronto, ON Canada; 2 Temerty Centre for Therapeutic Brain Intervention Centre for Addiction and Mental Health Toronto, ON Canada; 3 THETA and Biostatistics Unit University Health Network Toronto, ON Canada; 4 Campbell Family Mental Health Institute Centre for Addiction and Mental Health Toronto, ON Canada; 5 Aboriginal Engagement and Outreach Centre for Addiction and Mental Health Toronto, ON Canada; 6 Child and Youth Services Centre for Addiction and Mental Health Toronto, ON Canada; 7 Department of Psychiatry University of Toronto Toronto, ON Canada; 8 Centre for Addiction and Mental Health Toronto, ON Canada; 9 Mood and Anxiety Services Centre for Addiction and Mental Health Toronto, ON Canada; 10 Department of Psychology York University Toronto, ON Canada

**Keywords:** intervention study, telemedicine, mobile phone, mhealth, fitbit, depression, cognitive behavioral therapy

## Abstract

**Background:**

About 70% of all mental health disorders appear before the age of 25 years. When untreated, these disorders can become long-standing and impair multiple life domains. When compared with all Canadian youth (of different ages), individuals aged between 15 and 25 years are significantly more likely to experience mental health disorders, substance dependencies, and risks for suicidal ideation and death by suicide. Progress in the treatment of youth, capitalizing on their online responsivity, can strategically address depressive disorders.

**Objective:**

We will conduct a randomized controlled trial to compare online mindfulness-oriented cognitive behavioral therapy (CBT-M) combined with standard psychiatric care versus psychiatric care alone in youth diagnosed with major depressive disorder. We will enroll 168 subjects in the age range of 18 to 30 years; 50% of subjects will be from First Nations (FN) backgrounds, whereas the other 50% will be from all other ethnic backgrounds. There will be equal stratification into 2 intervention groups (INT^1^ and INT^2^) and 2 wait-list control groups (CTL^1^ and CTL^2^) with 42 subjects per group, resulting in an equal number of INT^1^ and CTL^1^ of FN background and INT^2^ and CTL^2^ of non-FN background.

**Methods:**

The inclusion criteria are: (1) age 18 to 30 years, FN background or other ethnicity; (2) Beck Depression Inventory (BDI)-II of at least mild severity (BDI-II score ≥14) and no upper limit; (3) Mini-International Neuropsychiatric Interview (MINI)–confirmed psychiatric diagnosis of major depressive disorder; and (4) fluent in English. All patients are diagnosed by a Centre for Addiction and Mental Health psychiatrist, with diagnoses confirmed using the MINI interview. The exclusion criteria are: (1) individuals receiving weekly structured psychotherapy; (2) individuals who meet the Diagnostic and Statistical Manual of Mental Disorders criteria for severe alcohol/substance use disorder in the past 3 months, or who demonstrate clinically significant suicidal ideation defined as imminent intent, or who have attempted suicide in the past 6 months; and (3) individuals with comorbid diagnoses of borderline personality, schizophrenia, bipolar disorder, and/or obsessive compulsive disorder. All subjects are provided standard psychiatric care defined as 1 monthly session that focuses on appropriate medication, with session durations of 15 to 30 min. Experimental subjects receive an additional intervention consisting of the CBT-M online software program (in collaboration with Nex J Health, Inc). Exposure to and interaction with the online workbooks are combined with navigation-coaching delivered by phone and secure text message interactions.

**Results:**

The outcomes selected, combined with measurement blinding, are key features in assessing whether significant benefits regarding depression and anxiety symptoms occur.

**Conclusions:**

If results confirm the hypothesis that youth can be effectively treated with online CBT-M, effective services may be widely delivered with less geographic restriction.

**International Registered Report Identifier (IRRID):**

PRR1-10.2196/11591

## Introduction

A total of 70% of all mental health problems appear before the age of 25 years. If untreated, these problems can become long-standing disorders that impair multiple life domains [1]. When compared with all Canadian youth (of different ages), the cohort between 15 and 25 years is significantly more likely to experience mental health disorders and substance dependencies and risks for suicidal ideation and death by suicide [2-5]. The current economic costs of mental health are vast, estimated at Can $51 billion annually, with Can $20.7 billion annually due to lost labor force participation [6]. Innovations in internet and smartphone technologies provide opportunities to deliver mental health care in ways that improve outcomes, reduce costs and overcome the geographic barriers that obstruct service equity.

Cognitive behavioral therapy (CBT)—the best-validated psychotherapy [7]—has, in recent years, been integrated with mindfulness meditation (MM), resulting in strong evidence supporting their combined effectiveness [8-14]. Research with this combination, with student populations, by our group, has resulted in psychometric and neurophysiological [9-17] benefits in online single-arm and randomized controlled trials (RCTs). Another online intervention, tested in an RCT, demonstrated significant reductions in glycosylated hemoglobin blood levels in patients with type 2 diabetes [18-20].

Mindfulness interventions gained notice due to the efficacy and low costs of easily learned procedures that fostered attentional skills and present-time awareness ([21-31]; Kirk et al, in press). For over three decades, in-person mindfulness-based stress reduction and acceptance and commitment therapy programs [17-24,32] have reduced mental and physical symptoms, summating in a significant accumulation of evidence supporting their effectiveness in nonclinical [28] and clinically diagnosed populations [29-31]. A recent review of mindfulness-based cognitive behavioral therapy (CBT-M) suggests that internet-based approaches have effects similar to in-person approaches [33] with evidential support from a systematic review of studies published between 2000 and 2018 [33-43]. The reviewed studies (all RCTs) assessed internet-delivered CBT-M programs in terms of changes in anxiety and depression (as primary or secondary outcomes) in adults (mean age ≥18 years) with a clinical diagnosis of depression or anxiety (using the Diagnostic and Statistical Manual [DSM]-IV protocol) [34-43]. In this review of 11 RCTs, a mean Hedges’ g of −0.47 was calculated based on a 45.6% reduction in depression symptoms when compared with the control populations. These reductions are similar to those found in a systematic review reported by Karyotaki et al who calculated a mean Hedges’ g of −0.27 (for depressive symptom reduction) in online, self-guided treatments that solely implemented CBT [44].

Online programs, altogether, can track usage and transmit text or email prompts to motivate adherence, resulting in higher motivation levels [45-48]. Access to a virtual mindfulness-based navigator-coach at greater frequencies than possible with face-to-face communication [45-49], and improved outcomes. The RCT described here combines these features and assesses them with youth with diagnosed major depressive disorders.

## Methods

### Aim

The study aims to evaluate the efficacy of CBT combined with MM to treat youth (aged 18-30 years) diagnosed with major depressive disorder. We will enroll 168 subjects, where 50% of the subjects will be from First Nations (FN) background, while the other 50% will be from all other ethnic backgrounds, stratified in 2 intervention groups and 2 (wait-list) control groups (42 subjects per group, where INT^1^ and CTL^1^ are from FN background and INT^2^ and CTL^2^ are from non-FN background). The intervention groups will be compared with the control groups at baseline, 3 months (mid intervention), and 6 months (post intervention), using validated outcome measures.

### Recruitment and Randomization

Subjects are identified from the wait-lists for services at the Centre for Addiction and Mental Health (CAMH) and through contacts with multiple Toronto-based clinics. Eligible patients interact with a research coordinator who reviews and explains the study. Eligibility is ascertained in person followed by written consent before randomization. The identification of most subjects follows CAMH procedures where research coordinators identify potential participants by prescreening new clinic referrals and notifying the investigative team and the client’s clinician about potential study eligibility. The clinician then asks the client if she/he is willing to meet with a study team member to explore participation. Only when a client agrees, is she/he approached.

A biostatistician at the Department of Biostatistics at University Health Network (G Tomlinson) performed an electronic randomization of participants with study IDs to the different groups (INT^1^, INT^2^, CTL^1^, and CTL^2^). The information regarding each study ID with its respective group allocation was transferred onto cards and placed in individually sealed envelopes. After a participant had completed the baseline questionnaires, the research coordinator opened the next envelope in the series to determine participant group allocation and their respective study ID.

On the basis of a careful review of previously successful studies [38,39,41], we determined a sample size of 42 participants per group in 4 groups (total of n=168). Type I error was set at alpha=.05 and power at 80%. Our projected sample size of 168 participants is deemed more than adequate for the detection of small to medium effect size. With an anticipated drop-out rate of up to 20%, we will recruit 208 participants (54 per group).

#### Inclusion Criteria

The inclusion criteria are as follows: (1) age 18 to 30 years, FN background or any other ethnicity; (2) Beck Depression Inventory (BDI)-II of at least mild severity but no upper limit (BDI-II score ≥14) [50]; (3) Mini-International Neuropsychiatric Interview (MINI)-confirmed psychiatric diagnosis of major depressive disorder [51]; and (4) fluent in English. All patients are diagnosed by a CAMH physician, and the diagnoses are confirmed using the MINI interview administered at the screening visit [51].

#### Exclusion Criteria

The exclusion criteria are: (1) individuals who are currently receiving weekly structured psychotherapy; (2) individuals who meet the DSM-V criteria for severe alcohol/substance use disorder in the past 3 months, or who demonstrate clinically significant suicidal ideation defined as imminent intent, or who have attempted suicide in the past 6 months; and (3) individuals with comorbid diagnoses of borderline personality, bipolar disorder, schizophrenia, and/or obsessive compulsive disorder.

### Intervention

All subjects are provided standard psychiatric care, involving 1 monthly session that focuses on appropriate medication, with session durations from 15 to 30 min. Experimental subjects receive the additional intervention, consisting of a CBT-M software program (in collaboration with Nex J Health, Inc), which is accessed online. Interactions with the online workbooks is combined with navigation-coaching (total 24 hours duration), primarily delivered in phone and text message interactions. In addition, each participant is given a Fitbit-HR Charge 2 that assesses physical steps and 24-hour heart rate in 5-second (averaged) durations (the software permits daily monitoring). The intervention content builds on 2 prior successful Web-based CBT-mindfulness RCTs with students [9,10,13,15-17] and on effective methods with other populations demonstrated in previous RCTs [52-55]. The online workbook content includes 24 chapters reflecting multiple topics (eg, Living By Your Truths, Overcoming Wired-ness and Tired-ness, Mindfulness and Relationships, Loss and Grief, and Resilience, Befriending Ourselves, Befriending Your Body with Exercise, Body Image and Mindfulness, Intimacy, Forgiveness, Overcoming Procrastination, Dealing with Negative Moods, Stress Resilience, Overcoming Performance Anxiety, and Cultivating Inspiration) covered sequentially on a weekly basis with the navigator-coach. In summary, the key intervention features are 24-hour access and CBT-mindfulness contents that address specific symptoms and generic depressive experiences.

### Hypothesis

The CBT-M online intervention will be associated with statistically and clinically significant between-group differences (benefits) when treatment groups and control groups are compared, using both intention-to-treat (ITT) and per protocol analyses (PP). The ITT will proceed in a standard manner, whereas the PP will be based on the 24-week and 24-session structure of the intervention. All subjects who fail to attend 50% of the sessions (ie, <12 sessions) will be excluded from the PP.

### Outcome Measures

#### Primary Outcome

The primary outcome measure is the BDI-II [50].

#### Secondary Outcomes

The secondary outcomes assess anxiety (Beck Anxiety Inventory) [56], depression (ie, Quick Inventory of Depressive Symptomatology) [57], 24-item Hamilton Depression Rating Scale (HDRS-24; with a blinded interview-rater) [58], mindfulness (5-Facet Mindfulness Questionnaire) [59], and pain (Brief Pain Inventory) [60].

All self-report measures and the HDRS-24 interview are carried out at the same CAMH Mood and Anxiety research clinic in identical assessment rooms. The HDRS-24 interview-rater is blinded to intervention and control conditions for the trial duration.

## Results

### Analyses

Data obtained from participants during the study visits are de-identified and stored as electronic case reporting forms (CRFs) on the CAMH REDCap system and physical CRF paper copies in a locked cabinet. Participant characteristics are summarized via descriptive statistics. Group equivalence at baseline in terms of demographic and clinical variables is assessed.

### Primary and Secondary Outcomes

The monthly rate of recruitment is calculated and the level of retention will be presented in the proportion of enrolled participants completing study outcomes at each time point.

The effect of the intervention on the primary clinical and secondary outcomes will be assessed through separate analysis of covariance (ANCOVA) models that have changed from baseline to 6 months as the dependent variable, the baseline value of the outcome as a covariate and the group assignment as a categorical variable. The treatment effect, its effect size (Hedges’ g), and 95% CIs for the treatment effect and within-group changes from baseline to one year will be calculated from the ANCOVA model. The sensitivity of results to missing data will be evaluated by running a purely data-based multiple imputation procedure, as well as the imputation of missing values on a case-by-case basis using expert opinion and patient history.

## Discussion

If hypothesized results are obtained, this intervention may be an important option for depressed youth. As it can be accessed wherever internet-based services are available, geographic barriers to high-quality treatment could be minimized. Acknowledged study limitations include the lack of blinding regarding administration of self-report measures other than the blinding maintained for the HDRS-24 assessment.

## Abbreviations

ANCOVA: analysis of covariance
BDI: Beck Depression Inventory
CAMH: Centre for Addiction and Mental Health
CBT: cognitive behavioral therapy
CBT-M: mindfulness-based cognitive behavioral therapy
CRF: case reporting form
DSM: Diagnostic and Statistical Manual of Mental Disorders
FN: First Nations
HDRS-24: 24-item Hamilton Depression Rating Scale
ITT: intention-to-treat
MINI: Mini-International Neuropsychiatric Interview
MM: mindfulness meditation
PP: per protocol
RCT: randomized controlled trial
